# Importance of Achieving a “Fit” Cardiorespiratory Fitness Level for Several Years on the Incidence of Type 2 Diabetes Mellitus: A Japanese Cohort Study

**DOI:** 10.2188/jea.JE20160199

**Published:** 2018-05-05

**Authors:** Haruki Momma, Susumu S. Sawada, Robert A. Sloan, Yuko Gando, Ryoko Kawakami, Shin Terada, Motohiko Miyachi, Chihiro Kinugawa, Takashi Okamoto, Koji Tsukamoto, Cong Huang, Ryoichi Nagatomi, Steven N. Blair

**Affiliations:** 1 Division of Biomedical Engineering for Health and Welfare, Tohoku University Graduate School of Biomedical Engineering, Sendai, Japan; 2 Department of Health Promotion and Exercise, National Institutes of Biomedical Innovation, Health and Nutrition, Tokyo, Japan; 3 Department of Psychosomatic Internal Medicine, Graduate Medical and Dental School, Kagoshima University, Kagoshima, Japan; 4 Faculty of Sport Sciences, Waseda University, Tokorozawa, Japan; 5 Department of Life Sciences, Graduate School of Arts and Sciences, The University of Tokyo, Tokyo, Japan; 6 Tokyo Gas Health Promotion Center, Tokyo, Japan; 7 Department of Epidemiology and Biostatistics, Arnold School of Public Health, University of South Carolina, Columbia, SC, USA; 8 Department of Exercise Science, Arnold School of Public Health, University of South Carolina, Columbia, SC, USA

**Keywords:** Physical Activity Reference for Health Promotion 2013, maximal oxygen uptake, area under the curve, metabolic memory

## Abstract

**Background:**

The “Physical Activity Reference for Health Promotion 2013” provides “fit” reference values for cardiorespiratory fitness (CRF) for good health. The importance of achieving a fit CRF level for several years on the subsequent prevention of type 2 diabetes mellitus (T2DM) remains to be clarified.

**Methods:**

This cohort study was conducted in 2,235 nondiabetic males aged 21 to 59 years, enrolled in April 1986 through March 1987. We calculated the ratio of the area under the curve (AUC_ratio_) for actual measured values and the AUC for the reference values of CRF in each individual during an 8-year measurement period before the baseline. According to whether they met a fit CRF level or not, participants were categorized into groups based on the AUC_ratio_ (Fit_AUC_ or Unfit_AUC_) and initial CRF (Fit_initial_ or Unfit_initial_), respectively. T2DM was evaluated on health checkups until March 2010.

**Results:**

During the follow-up period, 400 men developed T2DM. After adjustment for confounders, as compared with those in the Fit_AUC_ group, the hazard ratio (HR) for those in the Unfit_AUC_ group was 1.33 (95% confidence interval [CI], 1.06–1.65). A combined analysis with the categories of initial value and AUC_ratio_ showed that, compared with the Fit_initial_ and Fit_AUC_ group, the HRs of Fit_initial_ and Unfit_AUC_, Unfit_initial_ and Fit_AUC_, and Unfit_initial_ and Unfit_AUC_ groups were 1.41 (95% CI, 0.99–2.00), 1.18 (95% CI, 0.81–1.70), and 1.40 (95% CI, 1.08–1.83), respectively.

**Conclusion:**

Achievement of a fit CRF level established in the Japan physical activity guideline for several years was associated with lower subsequent risk of T2DM.

## INTRODUCTION

According to the International Diabetes Federation, the number of patients with diabetes has reached 415 million worldwide.^1^ Strategies to prevent or delay diabetes are needed to mitigate the projected increase to 642 million before 2040. Physical inactivity is widely known as one of the modifiable risk factors for the development of type 2 diabetes mellitus (T2DM).^2^ Cardiorespiratory fitness (CRF) is considered an objective measurement of physical activity level,^3^^–^^5^ and CRF level predicts the development of T2DM.^6^^–^^12^ Therefore, maintaining higher CRF is an important strategy to prevent T2DM.

In Japan, the Ministry of Health, Labor and Welfare published the “Physical Activity Reference for Health Promotion 2013” in March 2013.^13^ It describes the reference values of physical activities and CRF to be achieved by individuals to reduce the risks of various diseases, including diabetes. The reference value for CRF is established according to sex and age. These reference values are based on the results of a systematic review and meta-analysis of findings obtained on the association between non-communicable diseases and CRF.^13^ However, few studies have verified their validity,^14^^,^^15^ and there has only been one validation study examining T2DM as an outcome.^14^ Previously, we examined the association between CRF level and development of T2DM among Japanese males.^14^ We reported that those with a “fit” CRF level (greater than or equal to the reference value) at baseline had a lower risk of T2DM compared to those with an “unfit” CRF level (less than the reference value) at baseline.^14^ Although this finding suggests that a fit CRF level has a beneficial influence on reducing the risk of T2DM, the importance of achieving a fit CRF level for a given time period on the subsequent development of T2DM remains to be clarified.

We hypothesized that those who did not achieve a fit CRF level for several years would have a higher subsequent risk of T2DM. We further hypothesized that, even if CRF level temporarily exceeds the fit reference value, the subsequent risk of T2DM may increase unless the level is maintained for several years. To verify our hypotheses, we examined the association between achievement of a fit CRF level for several years and subsequent risk of T2DM in a cohort study among Japanese males.

## METHODS

### Participants

This cohort study investigated the relationship between CRF and health outcomes in Japanese men.^7^^,^^16^^,^^17^ Male employees of a company in the Tokyo area participated in this study. Annual medical examinations and exercise tests are conducted in the company to maintain the health of the employees in accordance with the Industrial Safety and Health Law and related laws in Japan.

A total of 9,221 men who underwent exercise tests and had annual medical examinations from April 1986 through March 1987 participated in this study. Of these, 2,488 males underwent a blood glucose test according to the Industrial Safety and Health Act and related laws in Japan. Moreover, to obtain the CRF levels during multiple measurement of CRF, men who performed four or more exercise tests during April 1979 through March 1987 were selected as follow-up participants. Men with fewer than four measurements were excluded (*n* = 124). Men whose accurate CRF values were impossible to estimate because of their inability to continue the exercise test for longer than 4 min due to abnormal electrocardiograms (ECGs) were also excluded (*n* = 6). Women were excluded from the study due to the small number of female employees in the company (*n* = 25). Men who had a cardiovascular disease (*n* = 1) or diabetes (*n* = 35) before 1986 were also excluded. All participants had no history of stroke before the baseline. Of the remaining 2,297 participants, 51 men who had left the company by the end of 1987, and 11 men with missing data from potential confounding factors were excluded. The final 2,235 men were selected as participants and were followed up until March 2010.

The ethics committee of the National Institutes of Biomedical Innovation, Health and Nutrition of Japan has approved this study.

### Medical examination

The medical examination performed in 1986 measured the study participants’ height, weight, and resting blood pressure. We measured body weight using a weighing scale, which was calibrated and standardized as per the law, and the participants were dressed lightly without shoes. We calculated body mass index (BMI) using the height and weight measurements. An automated sphygmomanometer measured the resting blood pressure with the participant in a sitting position. Furthermore, we investigated the potential confounding factors related to CRF and T2DM development of the participants, including drinking habits, smoking habits, working conditions, and family history of diabetes, using a self-administered questionnaire.

### Cardiorespiratory fitness

We used the estimated maximal oxygen uptake as an index of CRF. We measured the estimated maximal oxygen uptake with a submaximal exercise test using a bicycle ergometer. The exercise test was composed of a maximum of three stages, with each stage lasting 4 min, with the load gradually increasing with each stage. The load at the start of the test was 98 W, 86 W, 74 W, and 61 W for those 19 to 29, 30 to 39, 40 to 49, and 50 to 60 years old, respectively. We measured the heart rate from the R–R intervals on an ECG. We estimated the maximal heart rate based on the participant’s age and set the target heart rate at 85% of the maximal heart rate. The test increased the load by 37 W at each stage until the participant reached the target heart rate. If we detected an abnormal ECG, including an increased number of premature ventricular contractions during the exercise test, the test was terminated. We estimated the maximum oxygen uptake according to the Åstrand and Ryhming nomogram^18^ and the Åstrand age correction factors^19^ using the heart rate obtained in the final minute of the final stage for each participant. The method used to estimate the maximum oxygen uptake in this study was highly correlated with the direct method in a previous study (*r* = 0.92).^20^

**Figure 1.**
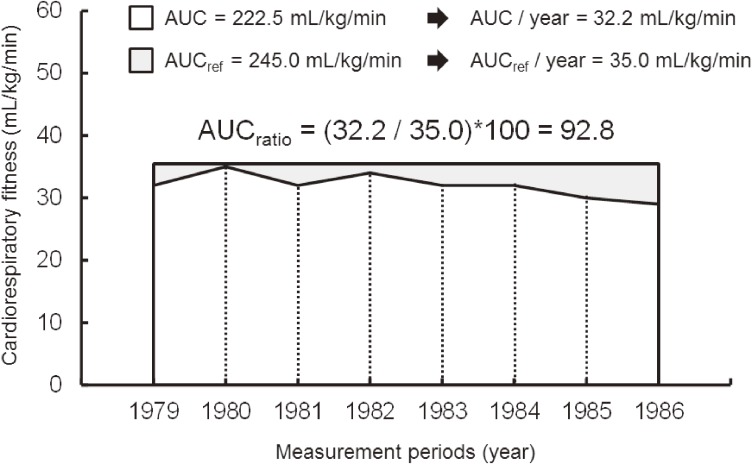
Scheme for calculating the area under the curve for cardiorespiratory fitness. The area under the curve (AUC) for cardiorespiratory fitness (CRF) was calculated based on a formula to calculate the trapezoid area. After calculating the area between measured values, the sum was calculated. Furthermore, based on the guideline for fit reference value,^13^ the reference area (AUC_ref_) for each individual during the same period was calculated. Next, we calculated the ratio of AUC to AUC_ref_ (AUC_ratio_) and the result was multiplied by 100 for intuitive interpretation. This figure shows the case of males aged 40 years old at 1979. The AUC and AUC_ref_ were divided by the measurement period (final year of measurement − first year of measurement), and standardized as the area per year.

To determine integrated CRF level from April 1979 through March 1987, we calculated the area under the curve (AUC) in each individual during the 8-year measurement period (Figure 1).^21^ Next, we calculated the reference area (AUC_ref_) for each individual based on the guideline for fit reference values during the same period. The reference values were provided by the guideline as follows: 39 mL/kg/min for males aged 18 to 39 years, 35 mL/kg/min for males aged 40 to 59 years, and 32 mL/kg/min for males aged 60 to 69 years.^13^ After calculating the ratio of AUC to AUC_ref_, we multiplied the result by 100 (AUC_ratio_). We assumed that those with the AUC_ratio_ above 100 achieved their CRF level above the reference values during the measurement period. If AUC_ratio_ was less than 100, we assumed that the CRF level was below the reference values during the period. Participants with an AUC_ratio_ of 100 or more were assigned to the Fit_AUC_ group, whereas participants with AUC_ratio_ less than 100 were assigned to the Unfit_AUC_ group. The number of CRF measurements during the 8-year measurement period differed for each participant; thus, we used the values of AUC and AUC_ref_ divided by the measurement period to standardize the indices. In addition to the AUC_ratio_ for CRF, the initial CRF measurement during the measurement period were also categorized into two groups: more than the reference values (Fit_initial_) or less than the reference values (Unfit_initial_) in accordance with the guideline.^13^

### Type 2 diabetes mellitus

During the medical examinations conducted from April 1986 through March 2010, we determined the year of T2DM development. From April 1986 and March 2010, participants with a fasting blood glucose level exceeding 7.0 mmol/L (126 mg/dL) were regarded as having diabetes. Furthermore, using a questionnaire administered during health checkups, we asked whether a diagnosis of T2DM was made by a physician. If more than one of the above criteria was met, the year of the earliest episode was regarded as the year of T2DM development.

### Statistical analysis

We compared baseline characteristics of participants based on CRF categories (Fit_AUC_ and Unfit_AUC_ groups). We showed the median value (interquartile range) for continuous variables and the percentage for category variables.

We set T2DM and the CRF category (Fit_AUC_ and Unfit_AUC_ groups) as the outcome and exposure variables, respectively. Cox proportional hazards regression analysis was used to compare the differences between T2DM incidence rates based on CRF categories, and calculated the age-adjusted hazard ratios (HRs) and 95% confidence intervals (CIs). Furthermore, we adjusted the following as potential confounding factors at baseline (1986): age (continuous variable), BMI (continuous variable), systolic blood pressure (continuous variable), smoking (never-smokers, past smokers, current smokers of 1–20 cigarettes/day, and ≥21 cigarettes/day), drinking habit (none, 1–40 g/day, and ≥41 g/day), desk work (yes or no), family history of diabetes (yes or no), fasting blood glucose (continuous variable), and frequency of measurements during the 8-year CRF measurement period (continuous variables). We also drew the adjusted cumulative incidence curve according to the category of the AUC_ratio_ for CRF using the above-mentioned multivariate model. We obtained the plot of one-minus-survival function by using the mean of each covariate as a constant value.

In addition, as the guideline described the CRF reference values according to age, we performed a stratified analysis, based on younger than 40 years and older than 40 years, by using the same model as the aforementioned covariates.

We also performed a sensitivity analysis, which excluded participants who had developed diabetes within 3 years of starting follow up, to investigate the effect of reverse causality.

Finally, a combination analysis was conducted using the categories based on the initial CRF measurement value (Fit_initial_ and Unfit_initial_) and the categories based on AUC for CRF (Fit_AUC_ and Unfit_AUC_ groups) during the measurement period. The Fit_initial_ group × Fit_AUC_ group was used as the reference group, and we calculated HRs and 95% CIs using the same covariates mentioned previously.

The proportionality assumption of the models was tested using a log-minus-log plot; no evidence of violation was found. All analyses were done using SPSS version 22 (IBM Japan, Tokyo, Japan). A two-tailed *P*-value less than 0.05 was considered statistically significant.

## RESULTS

### Study participants

The median age of the participants at the start of follow-up was 43 years. The median and maximum follow-up periods (April 1987 through March 2010) were 15 and 23 years, respectively. During total follow-up of 31,408 person-years, 400 participants developed T2DM. During follow-up before March 2010, a total of 1,636 participants dropped out. Of these, 46 dropped out at 49 years of age or younger for reasons other than mandatory retirement. The other 1,590 participants dropped out at 50 years of age or older possibly due to mandatory retirement.

Table 1 shows the characteristics of the study participants at baseline according to the category of the AUC_ratio_ for CRF. The BMI, systolic blood pressure, diastolic blood pressure, and fasting blood glucose at baseline (1986) in the Unfit_AUC_ group were higher than in the Fit_AUC_ group. In the Unfit_AUC_ group, the proportions of smokers, alcohol drinkers, those with a family history of diabetes, and those doing desk work were also higher than in the Fit_AUC_ group. On the other hand, the value of AUC_ratio_, initial CRF value between 1979 and 1986, and CRF at baseline (1986) in the Unfit_AUC_ group were lower than in the Fit_AUC_ group. The median frequency of CRF measurement between 1979 and 1986 was 7 times in the two groups.

**Table 1.** Baseline characteristics of participants according to the category based on the reference values of cardiorespiratory fitness

	All	Fit_AUC_	Unfit_AUC_
*n*	2,235	971	1,264
Age, years	43.0 (38.0–49.0)	44.0 (38.0–49.0)	43.0 (38.0–49.0)
BMI, kg/m^2^	23.2 (21.7–25.0)	22.4 (20.9–23.9)	24.0 (22.3–25.6)
SBP, mm Hg	129.0 (121.0–138.0)	127.0 (116.0–135.0)	131.0 (123.0–140.0)
DBP, mm Hg	75.0 (69.0–81.0)	73.0 (67.0–79.0)	76.0 (71.0–83.0)
Fasting blood glucose, mg/dL	93.0 (86.0–100.0)	92.0 (84.0–99.0)	94.0 (87.0–101.0)
Smoking status			
Never	680 (30.4)	303 (31.2)	377 (29.8)
Former	277 (12.4)	108 (11.1)	169 (13.4)
1–20/day	672 (30.1)	322 (33.2)	350 (27.7)
≥21/day	606 (27.1)	238 (24.5)	368 (29.1)
Drinking status			
None	556 (24.9)	236 (24.3)	320 (25.3)
1–40 g/day	1,526 (68.3)	679 (69.9)	847 (67.0)
≥41 g/day	153 (6.8)	56 (5.8)	97 (7.7)
Family history of diabetes	613 (27.4)	235 (24.2)	378 (29.9)
Desk work	784 (35.1)	312 (32.1)	472 (37.3)
Ratio of AUC to AUC_ref_, %	97.8 (89.1–107.2)	108.8 (103.8–115.9)	90.2 (84.6–95.2)
Initial CRF, mL/kg/min	35.5 (31.8–40.9)	40.0 (36.4–44.5)	32.7 (30.0–36.4)
CRF at baseline, mL/kg/min	36.0 (32.0–41.0)	41.0 (37.0–45.0)	34.0 (30.0–36.0)
Measurement times, times	7.0 (6.0–8.0)	7.0 (6.0–8.0)	7.0 (6.0–8.0)

AUC, area under the curve for cardiorespiratory fitness based on individuals’ measurements during 1979–1986; AUC_ref_, area under the curve based on the reference value of cardiorespiratory fitness in the text; BMI, body mass index; CRF, cardiorespiratory fitness; DBP, diastolic blood pressure; SBP, systolic blood pressure.

Data are presented as median (interquartile range) or *N* (%).

**Figure 2.**
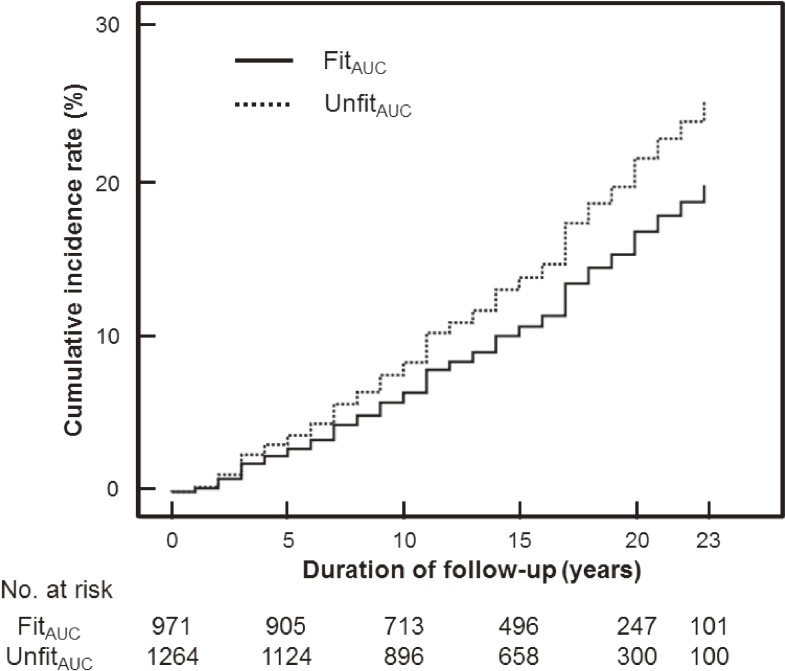
Multivariate-adjusted cumulative incidence curve of type 2 diabetes mellitus with respect to the category of the AUC_ratio_ for CRF. Covariates consisted of the age, BMI, systolic blood pressure, smoking (no smoking), alcohol drinking, deskwork, and family history of diabetes (presence or absence) at baseline (1986), and frequency of measurement during the 8-year measurement period of CRF. AUC, area under the curve; BMI, body mass index; CRF, cardiorespiratory fitness.

Table 2 shows the hazard ratio of T2DM according to the category of the AUC_ratio_ for CRF. The age-adjusted HR of T2DM in the Unfit_AUC_ group to that in the Fit_AUC_ group was 1.72 (95% CI, 1.39–2.12) (*P* < 0.001). Considering potential confounding factors, there was also a significant difference (HR 1.33; 95% CI, 1.06–1.65). Figure 2 shows the cumulative incidence curve after adjustment with confounding factors for T2DM according to the category of the AUC_ratio_ for CRF. In the Unfit_AUC_ group, the cumulative incidence of T2DM during the follow-up period was always higher than in the Fit_AUC_ group. In addition, a sensitivity analysis excluding participants who developed diabetes within 3 years after the start of follow-up showed that the multivariable-adjusted HR of T2DM in the Unfit_AUC_ group to that in the Fit_AUC_ group was higher (HR 1.28; 95% CI, 1.01–1.63).

**Table 2.** Hazard ratios for the incidence of type 2 diabetes according to the category based on the reference values of cardiorespiratory fitness

Category	*n*	Person-years of follow-up	Number of cases	Age-adjusted HR (95% CI)	Multivariate^a^ HR (95% CI)
Fit_AUC_	971	13,980	128	1.00 (Reference)	1.00 (Reference)
Unfit_AUC_	1,264	17,428	272	1.72 (1.39–2.12)	1.33 (1.06–1.65)
*P* value				<0.001	0.012

AUC, area under the curve for cardiorespiratory fitness based on individuals’ measurements during 1979–1986; CI, confidence interval; HR, hazard ratio.

^a^Adjusted for age (continuous variable), body mass index (continuous variable), systolic blood pressure (continuous variable), smoking status (never-smokers, former smoker, 1–20 cigarettes/day, and ≥21 cigarettes/day), drinking status (none, 1–40 g/day, and ≥41 g/day), a family history of diabetes (yes or no), desk work (yes or no), fasting glucose (continuous variable) at baseline, and measurement times of cardiorespiratory fitness (continuous variable).

As the reference values of CRF is established according to age, age-stratified analysis (<40 and ≥40 years) was performed (Table 3). Among participants with 40 years old or older, the multivariable-adjusted hazard ratios of T2DM in the Unfit_AUC_ group was higher in the Fit_AUC_ group (HR 1.40; 95% CI, 1.05–1.86). For participants with 39 years old or younger, the multivariable-adjusted hazard ratios of T2DM in the Unfit_AUC_ group was also higher, but not significantly, in the Fit_AUC_ (HR 1.28; 95% CI, 0.90–1.83).

**Table 3.** Hazard ratios for the incidence of type 2 diabetes according to the category of cardiorespiratory fitness stratified by age

Category	*n*	Person-years of follow-up	Number of cases	Multivariate^a^ HR (95% CI)	*P* value
Age 21–39 years					
Fit_AUC_	318	6,059	53	1.00 (Reference)	
Unfit_AUC_	433	7,902	112	1.28 (0.90–1.83)	0.18
Age 40–59 years					
Fit_AUC_	653	7,921	75	1.00 (Reference)	
Unfit_AUC_	831	9,526	160	1.40 (1.05–1.86)	0.023

AUC, area under the curve for cardiorespiratory fitness based on individuals’ measurements during 1979–1986; CI, confidence interval; HR, hazard ratio.

^a^Adjusted for age (continuous variable), body mass index (continuous variable), systolic blood pressure (continuous variable), smoking status (never-smokers, former smoker, 1–20 cigarettes/day, and ≥21 cigarettes/day), drinking status (none, 1–40 g/day, and ≥41 g/day), a family history of diabetes (yes or no), desk work (yes or no), fasting glucose (continuous variable) at baseline, and measurement times of cardiorespiratory fitness (continuous variable).

Table 4 shows the results of combined analysis with the categories of initial CRF value (Fit_initial_ and Unfit_initial_) and of AUC_ratio_ for CRF (Fit_AUC_ and Unfit_AUC_) during the measurement period based on the reference values. When a group in which both the initial CRF value and the AUC_ratio_ for CRF were higher than the reference levels (Fit_initial_ × Fit_AUC_) is set as a reference group, the multivariate-adjusted hazard ratios of T2DM in a group in which only the initial CRF value was higher than the reference levels (Fit_initial_ × Unfit_AUC_) and a group in which neither the initial CRF value nor the AUC_ratio_ for CRF was lower than the reference level (Unfit_initial_ × Unfit_AUC_) were 1.41 (95% CI, 0.99–2.00) and 1.40 (95% CI, 1.08–1.83), respectively. In a group in which only the AUC_ratio_ for CRF was higher than the reference level (Unfit_initial_ × Fit_AUC_), the HR was 1.18 (95% CI, 0.81–1.70).

**Table 4.** Hazard ratios for the incidence of type 2 diabetes according to the combined categories of cardiorespiratory fitness

Category	*n*	Person-years of follow-up	Number of cases	Age-adjusted HR (95% CI)	Multivariate^a^ HR (95% CI)
Fit_initial_ × Fit_AUC_	670	9,933	84	1.00 (Reference)	1.00 (Reference)
Fit_initial_ × Unfit_AUC_	253	3,917	52	1.59 (1.13–2.25)	1.41 (0.99–2.00)
Unfit_initial_ × Fit_AUC_	301	4,047	44	1.24 (0.86–1.79)	1.18 (0.81–1.70)
Unfit_initial_ × Unfit_AUC_	1,011	13,511	220	1.91 (1.48–2.45)	1.40 (1.08–1.83)

AUC, area under the curve for cardiorespiratory fitness based on individuals’ measurements during 1979–1986; CI, confidence interval; HR, hazard ratio.

^a^Adjusted for age (continuous variable), body mass index (continuous variable), systolic blood pressure (continuous variable), smoking status (never-smokers, former smoker, 1–20 cigarettes/day, and ≥21 cigarettes/day), drinking status (none, 1–40 g/day, and ≥41 g/day), a family history of diabetes (yes or no), desk work (yes or no), fasting glucose (continuous variable) at baseline, and measurement times of cardiorespiratory fitness (continuous variable).

## DISCUSSION

In this study, we examined the influence of achieving a fit CRF level (according to the Ministry of Health, Labour and Welfare guideline) for several years on the subsequent risk of T2DM among Japanese men. The results showed that those with an unfit CRF level during the measurement period (Unfit_AUC_) had a higher risk of T2DM compared with those with a fit CRF level during the period (Fit_AUC_). We also performed the combined analysis using the categories of initial CRF value and AUC_ratio_ for CRF during the period based on the reference values. As a result, even when the initial CRF level exceeded the fit reference value, the risk of T2DM in those who did not maintain a fit CRF level during the period was higher, but not significantly (*P* = 0.057), than that in those who maintained their CRF level over the fit reference values during the period. These results suggest that the achievement of a fit CRF level recommended in the “Physical Activity Reference for Health Promotion 2013” in Japan for several years contributes to a lower subsequent risk of T2DM.

Many previous studies have reported that a lower CRF level was associated with higher incidence of T2DM.^6^^–^^12^ This association has also been confirmed among Japanese males.^7^^–^^9^ For example, among Japanese males aged 20 to 40 years, the CRF level was negatively associated with the development of T2DM after a follow-up period of 14 years.^7^ Another study reported a negative association between physical performance in college and subsequent risk of T2DM.^8^ In line with these findings, we showed that, compared with those with an unfit CRF level according to the “Physical Activity Reference for Health Promotion 2013” in Japan at baseline, Japanese males with a fit CRF level had a lower risk of T2DM.^14^ This finding leads to a hypothesis that the risk of T2DM is reduced by achieving a fit CRF level recommended in the Japanese guideline over time. Based on this assumption, we examined the influence of achieving a fit CRF level for several years on the development of T2DM by calculating the ratio of the AUC for CRF to the reference area based on the reference values of the guideline during the 8-year measurement period for CRF. As a result, those with an unfit CRF level during the measurement period (Fit_AUC_) had a higher risk of T2DM compared to those with a fit CRF level (Fit_AUC_). The risk of incidence for T2DM in the Unfit_AUC_ category was 33% higher than that in the Fit_AUC_ category. This association was observed particular in participants aged more than 40 years. Therefore, these results suggested that the risk of T2DM could be reduced by achieving a fit CRF level recommended in the Japanese guideline for several years. In addition, even when the CRF level at baseline was above the fit reference values, the risk of T2DM was higher in participants with an unfit CRF level during the measurement period.

As a mechanism underlying the association between achievement of a fit CRF level for several years and lower incidence of T2DM, the cumulative effects of regular physical activities or exercise on glucose metabolism may be plausible. For example, animal studies showed that exercise during pregnancy in females enhanced insulin sensitivity and improved glucose homeostasis in mature offsprings.^22^^,^^23^ In addition, Shindo et al reported that rats that engaged in exercise during childhood had lower levels of blood glucose, insulin, and HOMA-IR at middle age after cessation of the exercise interventions compared with sedentary control rats.^24^ These findings may reflect the concept of “metabolic memory” or “cellular memory”, and changes in epigenetic modifications, including DNA demethylation, may be involved in its mechanism.^25^ In fact, previous human studies reported that acute exercise or exercise training for 6 months affected the methylation pattern of DNA or its promoter involved in glucose metabolism.^26^^–^^28^ The results of these studies support that epigenetic modification is a plausible mechanism explaining the association between achievement of a fit CRF level for several years and lower subsequent incidence of T2DM.

Interestingly, compared with the Fit_initial_ × Fit_AUC_ group, the risk of T2DM in the Unfit_initial_ × Fit_AUC_ group was higher, but not significantly, while the hazard ratio of the Unfit_initial_ × Fit_AUC_ group was lower than that of the Unfit_initial_ × Unfit_AUC_ group. This finding suggests the possibility that disadvantage of initial unfit CRF level that led to the development of T2DM was attenuated via the improvement of a fit CRF level recommended by the Japanese guideline after the initial CRF measurement. Therefore, health care professionals may thereby encourage those with an unfit CRF level to engage in physical activity for the prevention of T2DM. Further studies are necessary to examine the influence of duration or timing of meeting the reference values in CRF on T2DM.

This study has several limitations. First, the participants of this study were limited to males aged 21 to 59 years. Because previous studies reported that there was a negative association between CRF and T2DM even in a population including females and 79-year-old or younger males,^12^^,^^29^ whether or not the results of this study may be applied to females or elderly persons needs to be further examined. Second, we had small number of obese participants in the study population; those with BMI of ≥30 at baseline accounted only for 1% of the population. Therefore, the results of this study may be limited to a population with a relatively normal weight. Third, in this study, some potential confounding factors were considered, but the influence of unmeasured confounding factors, such as diet, was not ruled out. For example, it is possible that highly fit persons may have had more healthy dietary habits than low-fitness persons. Therefore, the relationship between CRF level and the incidence of T2DM may be attenuated by considering the influence of diet. Fourth, we could not precisely explain the reasons why individuals who did not achieve the fit CRF level at the initial measurement subsequently achieved the fit level, because we did not have any data of medical checkups and lifestyles at the initial measurement of CRF. Generally, it is well-known that moderate-vigorous physical activity or relatively high intensity exercise, such as endurance training, can improve the level of CRF. Lastly, whether individuals met a fit CRF level or not was considered only from April 1979 through March 1987, but not during the entire follow-up period from April 1987 through March 2010. Therefore, the influence of changes of CRF during the follow-up periods on our findings was not considered. The favorable influence of meeting a fit CRF level on the incidence of T2DM would be attenuated when secular change of CRF was considered.

In conclusion, our findings showed that the achievement of a fit CRF level recommended in the “Physical Activity Reference for Health Promotion 2013” in Japan was associated with lower subsequent risk of T2DM. These results suggested that the reference CRF values established in the Japanese guideline appear to be reasonably valid for prevention of T2DM among middle-aged Japanese males.
